# Cancer stem cells and tumor metastasis

**DOI:** 10.3892/ijo.2014.2362

**Published:** 2014-04-02

**Authors:** SHUANG LI, QIN LI

**Affiliations:** 1 The Southern Medical University, General Hospital of Guangzhou Military Command, Guangzhou, P.R. China; 2 Department of Plastic and Reconstructive Surgery, General Hospital of Guangzhou Military Command, Guangzhou, P.R. China

**Keywords:** cancer stem cells, transdifferentiation, lymphangiogenesis, angiogenesis, tumor metastasis

## Abstract

Previous studies have shown that tumors can induce angiogenesis and lymphangiogenesis, which plays an important role in promoting hematogenous and lymphogenous spread. In recent years, the cancer stem cell (CSC) theory has emerged as an attractive hypothesis for tumor development and progression. The theory proposes that one small subset of cancer cells has the characteristics of stem cells. These CSCs have the capability of both self-renewal and differentiation into diverse cancer cells, which play a decisive role in maintaining capacity for malignant proliferation, invasion, metastasis, and tumor recurrence. CSCs are involved in tumor metastasis, however, the details, and the possible relationship of CSCs, angiogenesis, lymphangiogenesis, and tumor metastasis is still ambiguous. The aim of this report is to summarize current studies of CSCs and tumor metastasis at the cellular level, with the goal of bringing new insights into understanding the role of CSCs in tumor metastasis.

## Contents

1. Introduction
2. Tumor-induced angiogenesis and lymphangiogenesis
3. The molecular mechanism of tumor-induced angiogenesis and lymphangiogenesis
4. The origin of tumor neogenetic endothelial cells
5. CSCs and tumor metastasis
6. CSCs and vasculature of tumor
7. CSCs and lymphatic vasculature as a significant thera peutic target
8. Summary and conclusions

## 1. Introduction

Metastasis is defined as the spread of cancer cells from the site of an original malignant primary tumor to one or more other places in the body. Over 90% of cancer suffering and death is associated with metastatic spread. Therefore, a significant aim of cancer research is to understand the molecular and cellular mechanisms that underlie the processes of metastasis.

Metastasis is most often associated with solid tumors. Evidence suggests that an important initial event in the spread of solid tumors is through the lymphatic system (lymphanogenous spread), while spread via blood vessels (hematogenous spread) may be secondary (1). Indeed, metastatic spread to regional lymph nodes is considered a prognostic indicator and may help to determine cancer management and therapy (1).

It is widely accepted that angiogenesis is involved in solid tumor growth and hematogenous spread. However, current opinion indicates that lymphangiogenesis plays the key role in promoting the initial spread of malignant tumors. Despite this, the mechanism that underlies lymphatic spread and the role of lymphangiogenesis in tumor metastasis is not clear.

Recently, the cancer stem cell (CSC) theory has emerged as an attractive hypothesis for tumor development and progression. The theory suggests that tumors consist of subsets of cells with functional heterogeneity. In the CSC model, one small subset of cancer cells has the characteristics of stem cells. These CSCs have the capability of both self-renewal and differentiation into diverse cancer cells, which play a decisive role in maintaining capacity for malignant proliferation, invasion, metastasis, and tumor recurrence (2,3). Assuming CSCs are relatively refractory to the therapies developed to eradicate the non-stem cell component of tumors, the CSC model provides a theoretical basis for developing therapies that target the minority CSC population and presents a new perspective for the treatment of cancer (3).

So how do CSCs play a role in tumor metastasis and especially in lymphatic metastasis? Accumulating evidence suggests that CSCs closely correlate to tumor metastasis (1–5). This idea is supported by previous experimental observations including: a) CSCs can induce cancer metastasis through multiple pathways; b) angiogenesis and lymphangiogenesis are significant pathological changes in the process of tumor metastasis; and c) CSCs participate in angiogenesis and lymphangiogenesis directly and indirectly.

In this review we investigate the possible relationship between CSCs, tumor-induced lymphangiogenesis, and lymphatic metastasis in an attempt to reveal cellular mechanisms associated with metastatic spread, and to inform research on the development of approaches to block tumor lymphatic metastasis (1,4).

## 2. Tumor-induced angiogenesis and lymphangiogenesis

Tumor-induced angiogenesis and lymphangiogenesis play an important role in promoting tumor growth and metastasis (5). The continued tumor growth is often associated with neovascularization. Intratumoral hypoxia upregulates the expression of the vascular endothelial growth factor (VEGF) which induces angiogenesis, offering the necessary routes for cell dissemination, changing vascular integrity and permeability and even promoting intravasation and extravasation (6). Moreover, hypoxia selects a subpopulation of tumor cells with an invasive and metastatic phenotype that have the capacities of escaping from the primary tumors (7).

Lymphangiogenesis is also considered as a potential facilitator of cancer metastasis. Cancer cells move to the regional lymph nodes draining the primary tumor tissues promote tumor cells to migrate to local lymph nodes and even to distant organs (23). A number of studies have confirmed the association between lymphatic vessel density and survival rate in patients with different types of cancer. Schoppmann *et al* investigated invasive breast cancer by immunohistochemical staining for the lymphatic endothelial marker podoplanin and the vascular endothelial marker CD34, and showed that lymphatic microvessel density and lymphovascular invasion correlated with lymph node metastasis (8). Beasley *et al* analyzed samples from human head and neck cancers by immunohistochemical staining for the lymphatic endothelial marker LYVE-1, CD34, and the pKi67 proliferation marker, and quantified the lymphangiogenic growth factor, vascular endothelial growth factor C, by real-time polymerase chain reaction. Their study provided evidence that proliferating lymphatics occur in human malignant tumors, and they reported that a high intratumoral lymph vessel density was significantly correlated with cervical node metastases and an infiltrating margin of tumor invasion (9).

The lymphatic system is the primary conduit for initial metastasis in numerous types of solid tumors, including breast, colon, and prostate cancers. Metastatic spread is enhanced by increased lymphangiogenesis in and around the primary tumor (10,11). Tumor-induced lymphangiogenesis at the tumor periphery correlates with lymph node metastasis. Elucidating the interaction between lymphangiogenesis and tumor metastasis will bring us new insights into mechanisms of lymphatic metastasis.

## 3. The molecular mechanism of tumor-induced angiogenesis and lymphangiogenesis

Various lymphatic growth factors and vascular growth factors participate in regulating tumor-induced angiogenesis and lymphangiogenesis. Most of these factors are shown to have dual effects and interact with each other during angiogenesis and lymphangiogenesis making distinguishing difficult. It is believed that these factors are secreted by tumor cells, stromal cells, and inflammatory cells in the tumor microenvironment (12–14).

Vascular endothelial growth factors (VEGFs) stimulate angiogenesis and lymphangiogenesis by activating VEGF receptor (VEGFR) tyrosine kinases in endothelial cells. The major signaling pathways for lymphangiogenesis include secreting type glucoprotein VEGF-C and VEGF-D, which function through VEGFR-3 (Flt4) expressed on the surface of lymphatic endothelial cells (LECs). At present, the VEGF-C/ VEGF-D/VEGFR-3 pathway is the most understood pathway regulating lymphangiogenesis (15,16). However, Tammela *et al* showed that VEGFR-3 as a regulator of vascular network formation. Targeting VEGFR-3 may provide additional efficacy for anti-angiogenic therapies, especially towards vessels that are resistant to VEGF or VEGFR-2 inhibitors (17).

**Figure 1.**
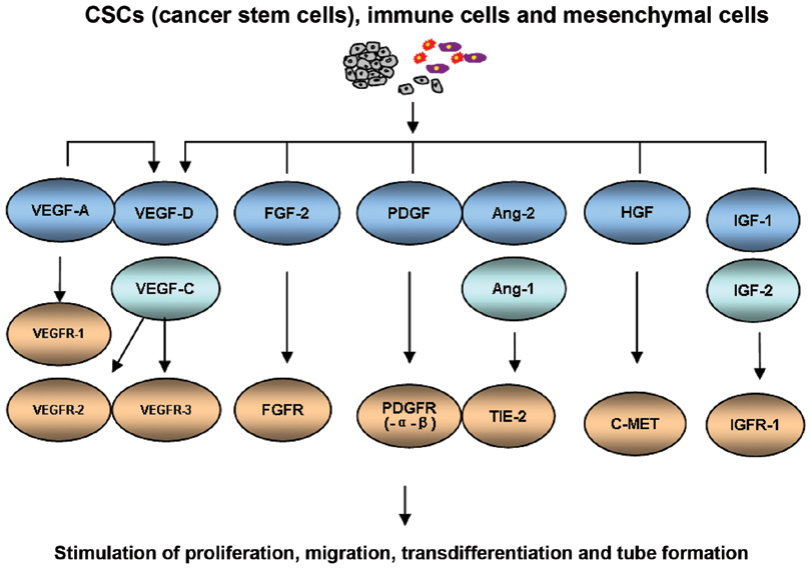
Lymphangiogenic growth factors and their receptors. Ang-1 and -2, angiopoietins-1 and -2; ET-1, endothelin-1; FGF-2, fibroblast growth factor-2; FGFR, fibroblast growth factor receptor; HGF, hepatocyte growth factor; IGF-1, -2, insulin-like growth factors-1 and -2; IGFR-1, insulin-like growth factor receptor-1; PDGF, platelet-derived growth factor; PDGFR, platelet-derived growth factor receptor; TIE-2, endothelial cell-specific receptor tyrosine kinase; VEGF-A, -C and -D, vascular endothelial growth factors-A, -C and -B; VEGFR-1, -2 and -3, vascular endothelial growth factor receptors-1, -2 and -3.

VEGF-A, another VEGF family member, initially identified as an important promoter of angiogenesis, primarily binds to VEGFR-1 and VEGFR-2 (18). However, VEGF-A can also induce tumor lymphangiogenesis and promote tumor metastasis to regional and distant lymph nodes (19). Tie-1,-2 and their ligand angiopoietins-1, -2 and -4 (Ang-1, -2 and -4) play a role in tumor angiogenesis and lymphangiogenesis. Ang/ Tie signaling pathway closely relates to the VEGF/VEGFR signaling pathway. VEGF-A, -C and PDGFB can upregulate Ang-2. Moreover, hepatocyte growth factor (HGF), fibroblast growth factor-2 (FGF-2), platelet-derived growth factor-BB (PDGFBB), insulin-like growth factors 1 and 2 (IGF-1 and -2), and endothelin-1 (ET-1) have been identified as inducers of angiogenesis and lymphangiogenesis. The main angiogenic and lymphangiogenic growth factors are summarized in Fig. 1.

CSCs show greater potential for angiogenesis and lymphangiogenesis than non-stem cell-like tumor cells. Malignant gliomas are highly lethal cancers dependent on angiogenesis. Bao *et al* (20) examined the potential of stem cell-like glioma cells (SCLGC) to support tumor angiogenesis. In comparison with matched non-SCLGC populations, SCLGC consistently secreted markedly elevated levels of vascular endothelial growth factor (VEGF), which were further induced by hypoxia. The VEGF expression in CD133^+^ SCLGC was 10–20-fold upregulated, combined with a dramatically increased vascular density identified by CD31 staining. In an *in vitro* model of angiogenesis, SCLGC-conditioned medium significantly increased endothelial cell migration and tube formation compared with non-SCLGC tumor cell-conditioned medium. Furthermore, therapy with VEGF neutralizing antibody (bevacizumab) can deplete SCLGC-induced vascular endothelial cell migration and tube formation. These data indicate that stem cell-like tumor cells can be a crucial source of key angiogenic factors in cancers and that targeting proangiogenic factors from stem cell-like tumor populations may be critical for patient therapy. Other studies support this finding in CSCs isolated from U87 glioma human cell lines and GL261 murine glioma cell lines (21,22). Additionally it was reported that CSCs contribute to tumor vascular development in glioma. The results revealed that tumor with larger CSC population recruited a substantially higher amount of endothelial progenitor cells (EPC), suggesting that CSCs promote local angiogenesis and EPC mobilization via stimulating proangiogenic factors such as VEGF and SDF-1 (23).

Related research has explored this phenomenon using high throughput assays in liver cancer, qRT-PCR assessment and tissue microarray (TMAs) validation (24). In the high hepatic stem/progenitor cell (HSC/HPC) profile group (CD133, Nestin CD44 and ABCG2), the MVD and angiogenic factors (VEGF and PD-ECGF) are significantly higher than in the low HSC/ HPC profile group and related to a poor prognosis. Both stemness and angiogenesis associated factors might be potential biomarkers for clinical prediction (24).

Moreover, recent research showed that there are signaling pathways associated with CSCs and angiogenesis. One is bone morphogenic protein (BMP) signaling. The BMP was shown to play a vital role in CSC tumorigenesis and angiogenesis (25,26). Another important mechanism is the Notch signaling pathway. Recently, Hovinga *et al* showed that the Notch pathway combines glioblastoma angiogenesis and cancer stem cell self-renewal (27). These two pathways are both related to CSCs and angiogenesis, however, further experiments need to be done to show the fundamental cause and effect.

## 4. The origin of tumor neogenetic endothelial cells

Identifying the origin of tumor neovascularized blood endothelial cells (BECs) and lymphatic endothelial cells (LECs) can help elucidate the cellular mechanisms of angiogenesis and lymphangiogenesis.

Potential cellular origins of LECs include pre-existing vasculature as well as bone marrow-derived progenitor cells (BMDCs). A large body of evidence suggests that newly formed lymphatic vessels primarily arise from the pre-existing local vascular/lymphatic network during angiogenesis/lymphangiogenesis and hematogenous/lymphanogenous spread (28–30). However, as BM-derived vascular endothelial progenitor cells (EPCs) support the formation of new blood vessels during tumor angiogenesis, it is possible that BMDCs also contribute to the expansion of the lymphatic vasculature during tumor metastasis (28,31–33). These findings have recently been challenged by the study of De Palma *et al* (34), who demonstrated BM-derived hemopoietic cells (CD45^+^/CD11b^+^/CD31^−^/Tie2^+^) rather than EPC (CD31^+^), homed specifically to tumors, without any evidence of incorporation. The reason for such diametrically conflicting results remains unclear. Indeed, recent research indicates that lymphangiogenesis can occur from BM-derived lymphatic lineage cells and that BM-derived non-endothelial cells can transdifferentiate into tumor LECs under certain conditions. In a model of mouse inflammation after corneal transplant, Maruyama *et al* (35) demonstrated that CD11b^+^ macrophages infiltrated the corneal stroma, transdifferentiated into LECs, and integrated into existing lymphatic vessels. In addition, tumor-associated macrophages (TAMs) express the lymphatic marker VEGFR-3. However, the transdifferentiation of TAMs into LECs during tumorigenesis requires further investigation (36).

BM-derived mesenchymal stem cells (MSCs) may also contribute to tumor angiogenesis and lympahgiogenesis. MSCs can infiltrate tumors and may enhance breast cancer cell metastasis (37). Furthermore, MSCs have the ability to differentiate into endothelial cells (ECs) under certain conditions, and ECs and MSCs are able to transdifferentiate and interchange their phenotypes (37–40). Such transdifferentiation may be facilitated by the tumor microenvironment and could contribute to tumor progression.

Several studies have shown that LECs can arise by transdifferentiation from blood endothelial cells (BECs). During embryonic lymphangiogenesis, lymphatic endothelial precursor cells are derived from venous ECs in the cardinal vein. This population of venous ECs expresses transcription factors, including COUP-TFII, PROX-1 and SOX18, which regulate the transdifferentiation of venous ECs into LECs (41–43). Therefore, reactivation of a specific set of transcription factors in the adult under certain pathological conditions may regulate the differentiation of ECs by turning on the molecular program required for transition from a BEC to a LEC phenotype. In support of this concept, studies show that blood vessels express the lymphatic marker VEGFR-3 in some tumors (44,45). The expression of VEGFR-3 on BECs can increase angiogenic activation by the VEGF pathway and induce the LEC phenotype and may be indicative of a phenotypic transition between blood and lymphatic vessels. The key pathways that trigger the angiogenesis and lymphangiogenic switch during tumorigenesis are shown in Table I.

## 5. CSCs and tumor metastasis

Studies show that CSCs closely correlate with tumor metastasis (2,46). Pandit *et al* compared differentially expressed genes in cell lines of high (468LN) vs. low (468GFP) lymphatic metastatic ability to identify genes of potential clinical relevance. This approach revealed that 468LN cells have a higher proportion of cells with a CSC-like (CD44^+^/CD24^−^) phenotype, have a higher clonogenic potential, and a greater ability to survive, establish and grow in a foreign microenvironment, relative to 468GFP cells (47). Wakamatsu *et al* immunohistochemically examined the expression and distribution of representative CSC markers ALDH1, CD44, and CD133 from the primary tumor and the lymph node metastasis of gastric cancer. They showed ALDH1 positivity to be significantly higher in diffuse-type lymph node metastasis than in the primary tumor. They concluded that this CSC marker is important for tumor invasion and metastasis, and that CSCs can promote the heterogeneity and lymphatic metastasis of cancer (48).

Li *et al* (49) hypothesized that a single cancer cell can be considered a CSC as long as it can: a) develop into a tumor and b) its filial generation can inherit its biological features. They cultured CD133^+^ colorectal cancer monoplast cells *in vitro* and analyzed the invasive and metastatic capabilities of CD133^+^ single cell-derived progenies (SCPs) in a nude mouse model. They found that CD133^+^ SCPs were more likely to produced tumors after nude mice transplantation compared with CD133^−^ SCPs, and that CD133^+^ cells were heterogeneous in invasion and metastasis *in vitro* and *in vivo*. They concluded that colorectal CSCs constitute a diverse subpopulation.

To study the role of CSCs in the process of tumor metastasis, Brabletz *et al* (50) suggested the migrating cancer stem (MCS)-cell concept. They proposed that CSCs *in situ* can transform to MCS cells by epithelial-mesenchymal transition (EMT). Subsequently, the MCS cells disseminate and form metastatic colonies. In support of this concept, several studies have shown that cells possessing both the stem and tumorigenic characteristics of CSCs can be derived from human mammary epithelial cells (51,52).

## 6. CSCs and vasculature of tumor

It is believed that tumor metastasis is a complex multistep process, characterized by local invasion followed by intravasation of cancer cells into blood and lymphatic vessels (53). The intrinsic properties of the tumor itself and the tumor microenvironment are likely to be the main triggers that determine the ability of cancer cells to metastasize (54).

Several studies have shown that CSCs can promote angiogenesis and lymphangiogenesis in tumor metastasis. Angiogenic and lymphangiogenic factors are highly expressed by CSCs under conditions of hypoxia, which suggests that CSCs can indirectly promote angiogenesis and lymphangiogenesis during tumorigenesis and progression. Moreover, CSCs may directly participate in angiogenesis by transforming into tumor vasculogenic stem/progenitor cells or constructing a tumor microcirculation by developing vasculogenic mimicry without an endothelial pattern. Shen *et al* (55) isolated the CSC line 2C4 from the spleen of mice with leukemia. By subcutaneously implanting enhanced green fluorescent protein (GFP)-expressing transfected 2C4 cells into SCID CB17 mice, a xenotransplant tumor was formed. CSC-derived GFP^+^ endothelial-like cells were identified by green fluorescence in 10-mm diameter tumors. *In vitro*, the morphology of the CSCs was altered to elongated endothelial-like cells under conditions of hypoxia, and the expression of VEGFR2 was unregulated in the presence of cytokines, such as IL-13 and GM-CSF. The authors concluded that CSCs transdifferentiated to blood vessel ECs and were important for tumor vasculogenesis. Bussolati *et al* (56) isolated and cloned a population of breast tumor stem cells, which expressed the endothelial markers CD31, VEGFR2 and FVIII, when cultured in the presence of VEGF. The endothelial differentiated breast tumor stem cells acquired the ability to organize into capillary-like structures after 6 h in culture on Matrigel.

The ability of CSCs to participate in the development of endothelium directly contributes to understanding of the mechanisms of tumorigenesis and development. It challenges traditional theory on cancer and anti-angiogenesis therapy and emphasizes the potential of tumor lymphatic metastasis-resistant therapy.

## 7. CSCs and lymphatic vasculature as a significant therapeutic target

The source of tumor neolymphangiogenesis remains to be elucidated. We hypothesize that direct transdifferentiation of CSCs into LECs occurs during tumor lymphatic metastasis. Establishing the exact relationship of CSCs, lymphangiogenesis, and lymphatic metastasis will help to reveal the mechanisms of tumor metastasis at the cellular level, and create new challenges for future research.

Limitations of anti-angiogenic therapy have been reported (57,58). Although preclinical and clinical studies have established that anti-angiogenic therapies have antitumoral effects and survival benefits, there are studies showing that tumor cells can develop multiple mechanisms of resistance, which can increase tumor invasion and distant metastasis. A more significant therapeutic strategy could target both blood and lymphatic vessels to maximize anti-tumor and anti-metastasis effects.

The critical challenge of anti-lymphangiogenic therapy is to control metastatic disease after surgical removal of the primary tumor or inhibition by anti-angiogenic agents. Anti-lymphangiogenic therapies may help prevent both lymph node and distant organ metastasis by targeting lymphangiogenic growth factors, lymph node metastasis, and cellular mechanisms of differentiation and transdifferenatiation to LECs. Therefore, the simultaneous use of anti-angiogenic and anti-lymphangiogenic agents may improve current therapy.

It is well accepted that CSCs play a significant role in tumorigenesis, metastasis, and recurrence. More and more studies will focus on the role of CSCs in lymphangiogenesis, possibly revealing new targets for anti-CSCs and anti-lymphangiogenic therapy.

## 8. Summary and conclusions

**Figure 2.**
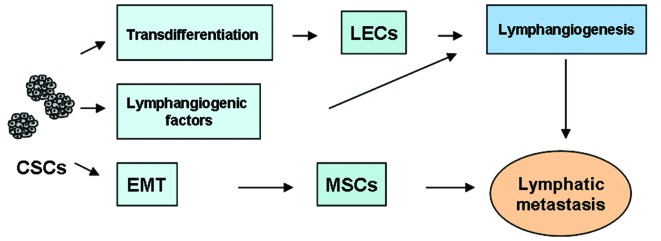
Proposed scheme for the relationship between cancer stem cells and lymphatic metastasis. CSCs, cancer stem cells; EMT, epithelial-mesenchymal transition; LECs, lymphatic endothelial cells; MSCs, mesenchymal stem cells.

**Figure 3.**
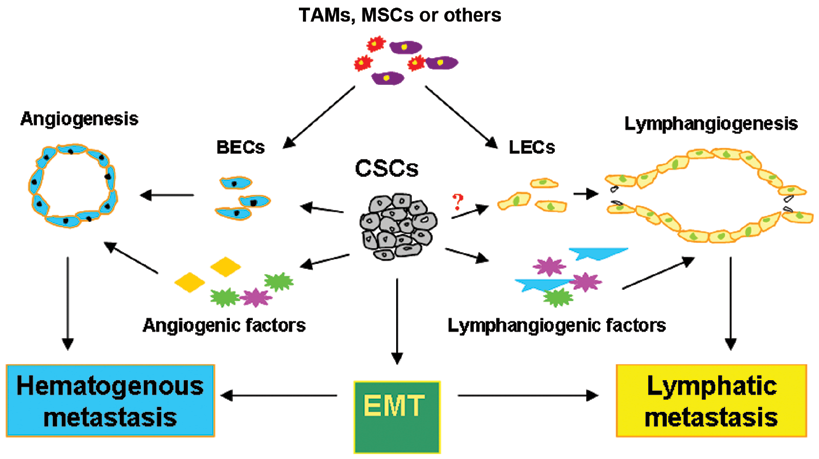
Proposed scheme for the relation of cancer stem cells and cancer metastasis. Blood endothelial cells in angiogenesis and lymphatic endothelial cells in lymphangiogenesis may be derived from different cells, including direct transdifferentiation from cancer stem cells. Cancer stem cells may also participate in angiogenesis and lymphangiogenesis through generating various angiogenic and lymphangiogenic factors. BECs, blood endothelial cells; CSCs, cancer stem cells; EMT, epithelial-mesenchymal transition; LECs, lymphatic endothelial cells; MSCs, mesenchymal stem cells; TAMs, tumor-associated macrophages.

In this review, we propose a relationship between CSCs and tumor metastasis. Clarification of this relationship may shed new light on cancer biotherapy. The multiple pathways through which CSCs can promote tumor metastasis are summarized in Figs. 2 and 3.

## Figures and Tables

**Table I.** Proposed cellular origin of lymphatic endothelial cells and blood endothelial cells.

Authors (Ref.)	Possible cellular origin	Main findings
De Palma *et al* (31) Lyden *et al* (32)	Bone-marrow-derived endothelial and hematopoietic precursor cells	Recruited to angiogenic sites and support the formation of new vessels
Maruyama *et al* (35)	Tumor-associated macrophages (TAMS)	Transdifferentiated into lymphatic endothelial cells that integrate into existing lymphatic vessels
Medici *et al* (40)	Bone marrow-derived mesenchymal stem cells (MSCs)	ECs and MSCs are able to interchange their phenotypes
Paavonen *et al* (44) Valtola R *et al* (45)	Blood endothelial cells (BECs)	The expression of VEGFR-3 on BECs in some tumors and chronic wounds
Shen *et al* (55) Bussolati *et al* (56)	Cancer stem cells (CSCs)	Transdifferentiation to blood vessel endothelial cells

## Abbreviations:

CSC: cancer stem cell;
LEC: lymphatic endothelial cell;
BEC: blood endothelial cells
